# Tissue Characteristics of Endometrial Carcinoma Analyzed by Quantitative Synthetic MRI and Diffusion-Weighted Imaging

**DOI:** 10.3390/diagnostics12122956

**Published:** 2022-11-25

**Authors:** Yiang Wang, Mengge He, Peng Cao, Philip P. C. Ip, Chien-Yuan Lin, Weiyin Liu, Chia-Wei Lee, Elaine Y. P. Lee

**Affiliations:** 1 Department of Diagnostic Radiology, The University of Hong Kong, Hong Kong SAR, China; 2 Department of Pathology, The University of Hong Kong, Hong Kong SAR, China; 3 GE Healthcare, Taipei 104, Taiwan

**Keywords:** endometrial carcinoma, synthetic magnetic resonance imaging, diffusion-weighted imaging, histopathologic features

## Abstract

Background: This study investigates the association of T1, T2, proton density (PD) and the apparent diffusion coefficient (ADC) with histopathologic features of endometrial carcinoma (EC). Methods: One hundred and nine EC patients were prospectively enrolled from August 2019 to December 2020. Synthetic magnetic resonance imaging (MRI) was acquired through one acquisition, in addition to diffusion-weighted imaging (DWI) and other conventional sequences using 1.5T MRI. T1, T2, PD derived from synthetic MRI and ADC derived from DWI were compared among different histopathologic features, namely the depth of myometrial invasion (MI), tumor grade, cervical stromal invasion (CSI) and lymphovascular invasion (LVSI) of EC by the Mann–Whitney U test. Classification models based on the significant MRI metrics were constructed with their respective receiver operating characteristic (ROC) curves, and their micro-averaged ROC was used to evaluate the overall performance of these significant MRI metrics in determining aggressive histopathologic features of EC. Results: EC with MI had significantly lower T2, PD and ADC than those without MI (*p* = 0.007, 0.006 and 0.043, respectively). Grade 2–3 EC and EC with LVSI had significantly lower ADC than grade 1 EC and EC without LVSI, respectively (*p* = 0.005, *p* = 0.020). There were no differences in the MRI metrics in EC with or without CSI. Micro-averaged ROC of the three models had an area under the curve of 0.83. Conclusions: Synthetic MRI provided quantitative metrics to characterize EC with one single acquisition. Low T2, PD and ADC were associated with aggressive histopathologic features of EC, offering excellent performance in determining aggressive histopathologic features of EC.

## 1. Introduction

Endometrial carcinoma (EC) is the most common gynecologic cancer in women [1]. The histopathologic features, such as depth of myometrial invasion (MI), tumor grade, cervical stromal invasion (CSI) and lymphovascular invasion (LVSI) affect the surgical management of EC and disease prognosis [2]. The depth of MI is correlated with the risk of recurrence and survival rates [3]. Therefore, non-invasive pre-operative evaluation of these histopathologic features will be valuable.

Given the high soft tissue resolution, magnetic resonance imaging (MRI) is recommended for the pre-operative assessment of EC. Diffusion-weighted imaging (DWI) has been explored to evaluate EC with encouraging results [4,5]. Aside from the apparent diffusion coefficient (ADC) from DWI, there are other metrics that offer quantitative tissue characterization, such as T1, T2, and proton density (PD) [6]. T1 and T2 quantification showed potential application in distinguishing tissue types and correlated with histopathologic features in different diseases, for example, in lung cancer and prostatic tissue [7,8]. However, conventional T1, T2 and PD-based quantitative MRI have not been explored to evaluate EC, mostly due to the long acquisition time with the individual sequences and consequently hampered routine clinical integration [9].

Recently, a novel synthetic MRI technique dubbed quantification of relaxation times and proton density by multi-echo acquisition of a saturation-recovery using the Turbo spin-Echo Readout (QRAPMASTER) method allows simultaneous measurements of T1, T2 and PD values, as well as generates different contrast-weighted images through a single acquisition [10]. QRAPMASTER is based on a 2D multi-dynamic, multi-echo (MDME) sequence which is performed using an interleaved slice-selective 120 degrees saturation and multi-echo acquisition. Parametric maps can be estimated by fitting the signal intensities of the reconstructed images generated from MDME with the relaxation curves modulated by specific T1, T2 and PD values on a voxel-by-voxel basis.

Synthetic MRI has shown promising clinical applications in generating parametric maps and multi-contrast images with reduced scan times and potential useful quantitative metrics. Compared with conventional quantitative MRI, QRAPMASTER reduces scan time, as well as producing high-quality synthetic contrast-weighted images [11]. It can also avoid misregistration, which is especially challenging in pelvic imaging. Synthetic MRI showed low T1, T2 and PD quantification errors overall, as well as high repeatability [12]. It has shown clinical application in neuroimaging, in the assessment of ischemic stroke, and demonstrated higher lesion-to-white matter with higher contrast-to-noise ratios in multiple sclerosis plaques [11,13,14]. In breast tissue, the quantitative metrics derived from synthetic MRI could differentiate benign and malignant breast lesions [15].

With these encouraging results, we hypothesize that synthetic MRI quantitative metrics could offer useful association with different histopathologic characteristics of EC. This study aimed to investigate the feasibility of deriving T1, T2 and PD of EC from synthetic MRI, and their association together with ADC from DWI in different histopathologic features of EC, namely MI, tumor grade, CSI and LVSI.

## 2. Materials and Methods

This prospective study was approved by the local institutional review board, and all study subjects gave informed consent.

### 2.1. Study Subjects Recruitment

**Figure 1:**
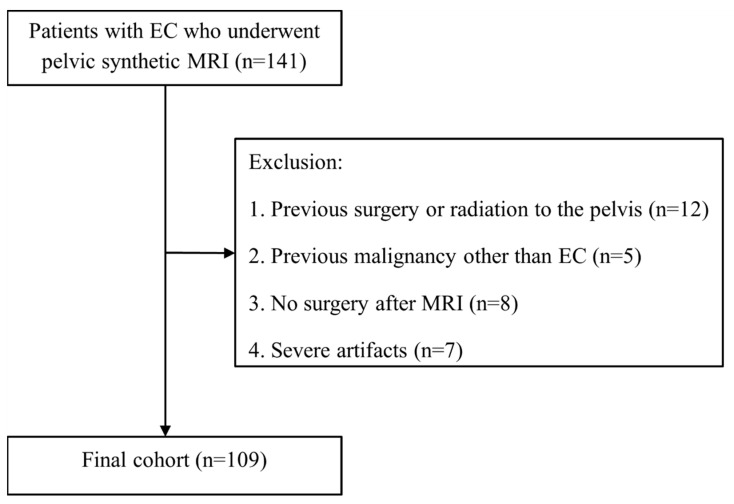
Flowchart of patient recruitment.

Consecutive patients with suspected EC were recruited from August 2019 to December 2020. The inclusion criteria included females (a) with histologically confirmed EC, and (b) who had undergone pre-operative MRI with synthetic MRI. Patients with (a) previous history of malignancy other than EC, (b) prior history of radiation to the pelvis or pelvic surgery, (c) severe artifacts observed on synthetic MRI images, and (d) who had not undergone surgery after MRI were excluded (Figure 1).

### 2.2. Image Acquisition and Post-Processing

All the MRI images were acquired using a 1.5 T MRI scanner (SIGNA Explorer, GE Healthcare, Waukesha, WI, USA) with a 16-channel body array coil. To reduce peristaltic artifacts, all the subjects fasted for 6 h and were given intravenous hyoscine butylbromide (Buscopan, Boehringer Ingelheim, Ingelheim am Rhein, Germany) before MRI examinations. Standardized MRI protocol, including conventional T2W, T1W, dynamic contrast-enhanced MRI (DCE-MRI), DWI (b = 0, 400, 800) and synthetic MRI, are summarized in Table 1.

The image post-processing was performed on a 64-bit Advantage Workstation (GE Healthcare, Waukesha, WI, USA), which generated the parametric maps (T1, T2, PD and ADC), as well as synthetic contrast-weighted images automatically. The mean of the T1/T2/PD/ADC maps were calculated inside the region-of-interest (ROIs) for all slices that contained the tumor.

### 2.3. MRI Interpretation

**Figure 2:**
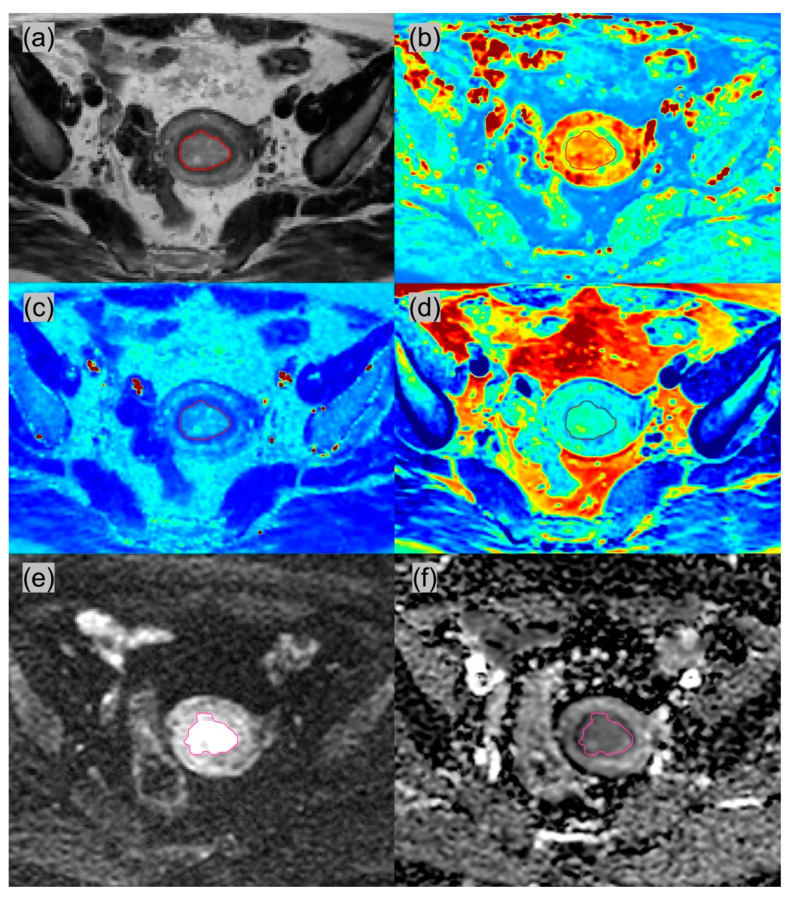
A 59-year-old woman with well-differentiated G1, FIGO stage IA endometrioid adenocarcinoma. The tumor had superficial myometrial invasion but without cervical stromal involvement or lymphovascular invasion. (**a**) Synthetic T2-weighted image; (**b**) T1 map; (**c**) T2 map; (**d**) PD map; (**e**) diffusion-weighted image with b = 800; (**f**) ADC map. The ROI was delineated on Synthetic T2WI and DWI. The mean value of all voxels within the ROIs: T1 = 1210.0 msec; T2 = 134.4 msec; PD = 86.1 pu; ADC = 1.201 × 10^−3^ mm^2^/s. PD, proton density; ADC, apparent diffusion coefficient.

**Figure 3:**
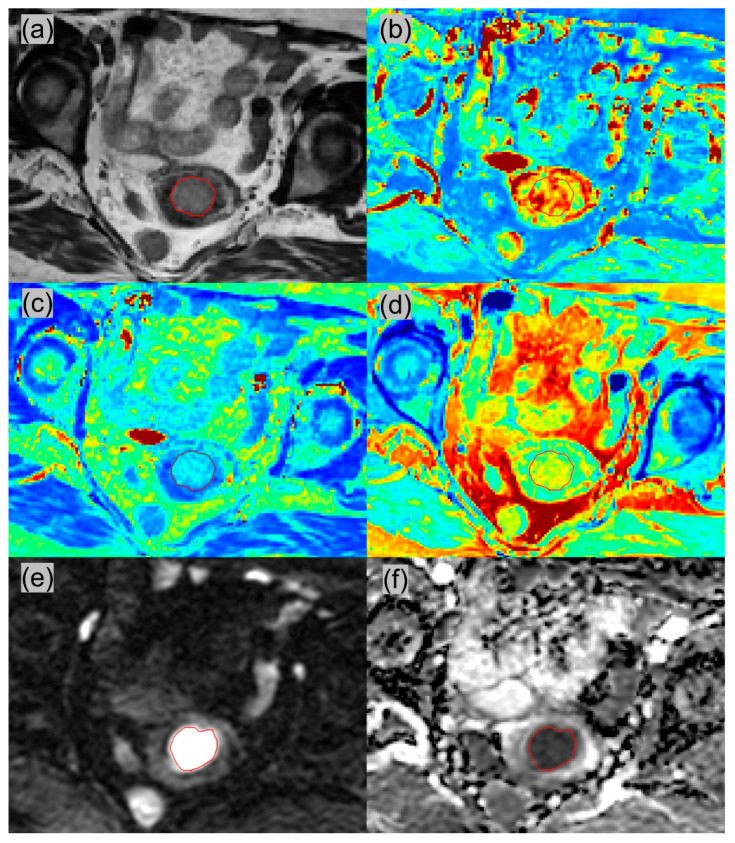
A 66-year-old woman with well-differentiated G2, FIGO stage II endometrioid adenocarcinoma. The tumor had superficial myometrial invasion, cervical stromal invasion and lymphovascular invasion. (**a**) Synthetic T2-weighted image; (**b**) T1 map; (**c**) T2 map; (**d**) PD map; (**e**) diffusion-weighted image with b = 800; (**f**) ADC map. The ROI was delineated on Synthetic T2WI and DWI. The mean value of all voxels within the ROIs: T1 = 1225.7 msec; T2 = 104.8 msec; PD = 85.7 pu; ADC = 0.913 × 10^−3^ mm^2^/s.

All MRI images were reviewed by two radiologists (1st radiologist with 4 years’ experience, and 2nd board-certified radiologist with >10 years’ experience in pelvic MRI). All slices with EC on synthetic T2W images was selected for ROI delineation. Similarly, the same was performed on DWI. The delineation was performed using the ITK-SNAP software (version 3.8.0). The T1, T2, PD and ADC values calculated within the ROIs were averaged from the two radiologists’ results and recorded for further statistical analysis. The ROI delineations between the two radiologists were visually compared, and if there was discrepancy in ROI delineations, this would be resolved in consensus before the values were averaged. The intraclass correlation coefficient (ICC) was calculated for each quantitative parameter to evaluate the interobserver agreement. Representative synthetic T2WI, DWI with b = 800, and parametric maps are shown in Figure 2 and Figure 3.

### 2.4. Histopathologic Assessment

All patients underwent hysterectomy with or without bilateral salpingo-oophorectomy. The surgical specimens were evaluated by an experienced pathologist specialized in gynecologic malignancy (>20 years’ experience). All cases were discussed at the local multi-disciplinary meeting.

### 2.5. Statistical Analysis

The statistical analyses were performed using R code (version 4.0.5) in R Studio. Age was represented as mean ± standard deviation, while the measured values (T1, T2, PD and ADC) were represented as median (95% CI). Histopathologic features were dichotomized (MI: no MI vs. MI; tumor grade: grade 1 (G1) vs. grade 2–3 (G2–3); CSI/LVSI: absent vs. present), while EC with MI were further dichotomized to superficial and deep MI. The Mann–Whitney U (MWU) test was used to test the differences between the median values of the two categories. A logistic regression model was built using MR metrics with significant differences (i.e., *p*-value < 0.05) for each histopathologic feature. The receiver operating characteristic (ROC) curve and the area under the ROC curve (AUC) were used to evaluate the classification performance of single-variable or multivariable models. Micro-averaged ROC and AUC were used to evaluate the overall performance of classifiers built for each histopathologic feature [16].

## 3. Results

### 3.1. Clinical Characteristics

One hundred and nine patients with EC (age 57.5 ± 9.6) were analyzed. The clinical characteristics are summarized in Table 2. The majority of cases had endometrioid adenocarcinoma (92.7%). For MI, 16.5% were without myometrial invasion, 61.5% were with superficial myometrial invasion and 22.0% with deep myometrial invasion. For tumor grades, 48.6% of tumors were classified as G1, 30.3% as G2 and 21.1% as G3. For FIGO stages, 75.2% of tumors were classified as stage I, 5.5% as stage II, 16.5% as stage III and 2.8% as stage IV. CSI and LVSI were absent in 89.0% and 82.2% of cases, respectively.

### 3.2. Interobserver Agreement of Quantitative Parameters

The ICCs for T1, T2, PD and ADC values between two radiologists were 0.961, 0.930, 0.904 and 0.960, indicating excellent interobserver agreements.

### 3.3. Association of MRI Metrics and Histopathologic Features

**Figure 4:**
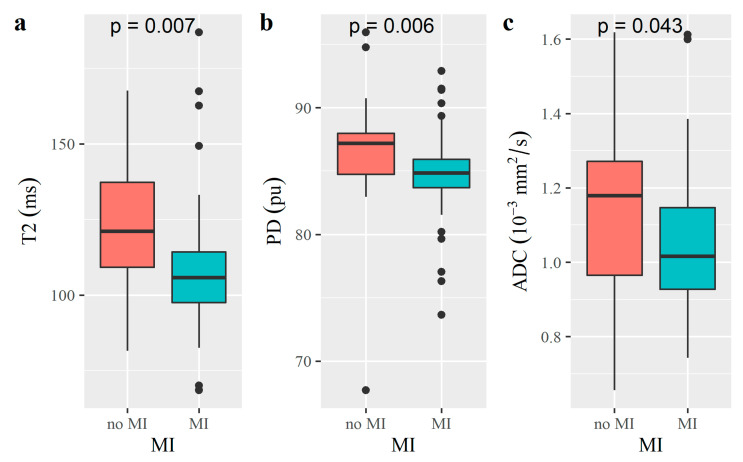
Boxplots of findings with representative significant differences (*p* < 0.05) of T2 (**a**), PD (**b**) and ADC (**c**) in patients with or without MI.

The differences of median T1, T2, PD and ADC values between different histopathologic features were summarized in Table 3. The T2, PD and ADC values of patients with MI were significantly lower than those without MI (*p* = 0.007, 0.006 and 0.043, respectively; Table 3 and Figure 4). There was no significant difference in T1, T2, PD or ADC values between patients with superficial MI and deep MI (*p* = 0.889, 0.739, 0.482 and 0.811, respectively; Table 3).

**Figure 5:**
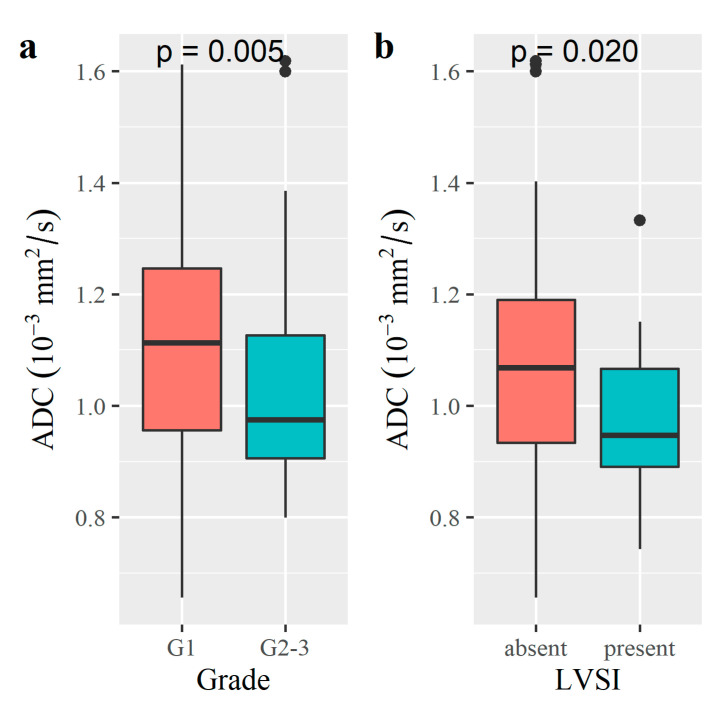
Boxplots of findings with representative significant differences (*p* < 0.05) of ADC in tumor grade (**a**) and LVSI (**b**).

Patients with a G2–3 tumor had significantly lower ADC values compared to those with a G1 tumor (*p* = 0.005, Table 3 and Figure 5). There was no difference in T1, T2 or PD values between G1 and G2–3 tumors.

Patients with LVSI had significantly lower ADC values compared to those without LVSI (*p* = 0.020, Table 3 and Figure 5).

No difference was observed in T1, T2, PD or ADC values in EC with and without CSI.

**Figure 6:**
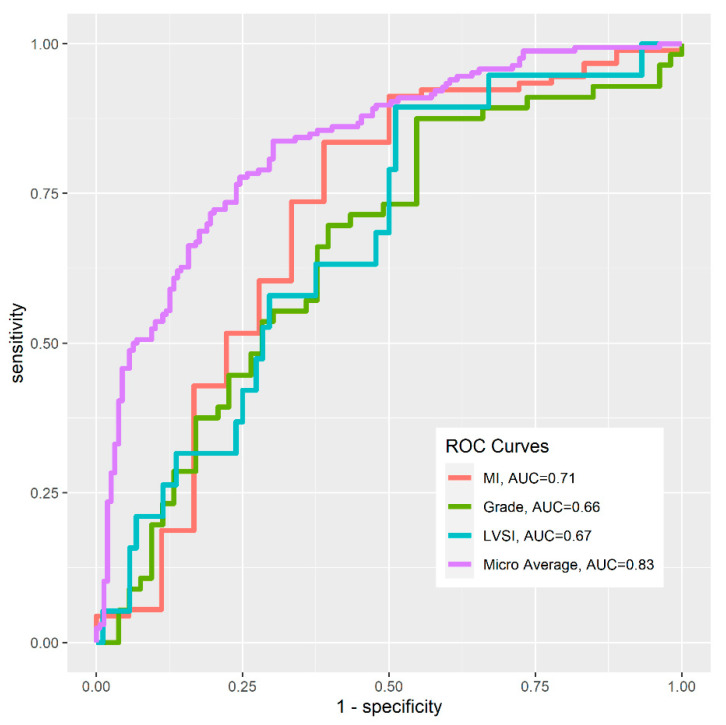
Receiver operating characteristic (ROC) curves for three classification models (MI, Grade and LVSI) and their micro-average.

Classification models were built for MI, tumor grades and LVSI (MI~T2 + PD + ADC; Grade~ADC; LVSI~ADC). The ROC curves of three classification models and the micro-averaged ROC curve were shown in Figure 6 (MI: AUC = 0.71; Grade: AUC = 0.66; LVSI: AUC = 0.67; Micro-average: AUC = 0.83).

## 4. Discussion

In this study, MR tissue properties measured by synthetic MRI through a single acquisition, including T1, T2 and PD values, as well as ADC measured by DWI, were evaluated in different histopathologic characteristics of EC. Our results showed that low T2, PD and ADC values were associated with more aggressive histopathologic features of EC with MI, higher tumor grades and LVSI.

In patients without MI, conservative management can be considered, especially for younger patients who wish to preserve fertility; for example, in patients under age 40 diagnosed with G1 endometrioid adenocarcinoma without evidence of MI and metastatic tumors, progestin therapy can be considered [2]. The association between MRI derived quantitative metrics and the depth of myometrial invasion offers additional information in pre-surgical assessment. However, similar to Rechichi et al. and Deng et al., we found no difference of ADC values between EC with deep and superficial MI [5,17].

In tumor grading, ADC values of G3 tumors were reported lower than that of G1 or G2 tumors [18,19,20]. Nevertheless, contradictory results were found in other studies in that ADC could not grade tumors in EC [4,17,21,22]. Herein, we found significantly lower ADC values of G2–3 tumors, in concordance with a previous study that had a cohort of 317 patients with EC [23]. In addition, the ADC values in EC with LVSI was significantly lower than those without, demonstrating the role of ADC in determining tumor aggressiveness.

In the prostate, T1 and T2 values of stromal hyperplasia were significantly higher than malignancy. In addition, prostate cancer and the non-cancerous peripheral zone could be differentiated by T1 and T2 values [24]. Gao et al. found that T2 values were useful in differentiating molecular subtypes of breast cancer [25]. In non-malignant conditions, T1, T2 and PD values were used to evaluate the severity of disc degeneration and Alzheimer’s disease [26,27]. Although synthetic MRI and quantitative T1 and T2 analyses have been used to assess various pathologic conditions, these have not been explored in EC. In the current study, T2, PD and ADC values were associated with MI, tumor grades and LVSI in EC, but not with CSI. Ye et al. found that high-risk EC was associated with lower perfusion metrics on dynamic contrast-enhanced MRI [28]. We speculate that less neovascularization in EC with more aggressive features might result in lower T2 and PD values, similar to observation in breast cancer [29]. A possible reason for the lack of association with CSI could be that a much larger sample size would be required to detect the small difference with 5% type I error and 80% power in a Mann–Whitney U test [30].

There were several limitations in this study. First, synthetic MRI was only acquired on the axial plane; hence, the generated weighted images could not be used in the qualitative evaluation, in which the oblique axial plane perpendicular to the long axis of the endometrial cavity is essential to allow accurate assessment of myometrial invasion. Second, the sample size was small, and subgroups were imbalanced, which might result in statistical bias and an insignificant difference between groups. Third, the T1, T2, and PD values derived from synthetic MRI were not compared with conventional MRI quantitative mappings, but the reliability of synthetic MRI has been validated on previous phantom and in vivo studies [12,13,27]. Fourth, although the histologic subtype is an important prognostic factor in EC, we were unable to perform further analysis as a vast majority of the cohort was affected by endometrioid adenocarcinoma, with only few who had other non-endometrioid subtypes. Fifth, EC tumor heterogeneity was not considered in this study, which might have affected the analysis. Further analysis with radiomics may offer insight. Finally, the study was based on data from one institution, which needs to be tested in independent datasets from other institutions.

In conclusion, low T2, PD derived from synthetic MRI, and ADC extracted from DWI were associated with aggressive histopathologic features of EC, specifically in MI, higher tumor grades and LVSI with excellent combined classification performance.

## Figures and Tables

**Table 1:** MRI Protocols for conventional MRI and synthetic MRI.

	T1WI	T2WI			DWI	DCE-MRI	Synthetic MRI
Sequence	Fast spin echo	PROPELLER			EPI	DISCO	QRAPMASTER
Plane	Axial	Sagittal	Coronal	Axial	Axial	Axial	Axial
Repetition time (msec)	603	8466	9943	9292	6831	6.6	4516
Echo time (msec)	7.8	125.4	125.6	115.4	70.6	3.1	19.3/86.8
FOV (cm)	35 × 35	24 × 24	26 × 26	24 × 24	35 × 35	24 × 24	35 × 35
Matrix Size	320 × 192	256 × 256	288 × 288	320 × 320	256 × 256	256 × 192	256 × 256
Slice Thickness (mm)	5.0	4.0	5.0	4.0	5.0	4.0	5.0
Bandwidth (kHz)	35.7	62.5	62.5	83.3	250	83.3	20.8
Number of excitations	2	3	1.5	2	2/4/8	1.18	1

MRI, magnetic resonance imaging; T1WI, T1 weighted imaging; T2WI, T2 weighted imaging; DWI, diffusion-weighted imaging; DCE-MRI, dynamic contrast-enhanced magnetic resonance imaging; PROPELLER, periodically rotated overlapping parallel lines with enhanced reconstruction; EPI, echo-planar imaging; DISCO, differential subsampling with cartesian ordering; QRAPMASTER, quantification of relaxation times and proton density by multi-echo acquisition of a saturation-recovery using turbo spin-echo readout; FOV, field of view.

**Table 2:** Clinical characteristics of patients with endometrial cancer.

Clinical Characteristics	Value	Percentage
Numbers in total	109	
Age	57.5 ± 9.6	
FIGO stages (*n*)	109	
I	82	75.2%
II	6	5.5%
III	18	16.5%
IV	3	2.8%
Histologic subtypes (*n*)	109	
Endometrioid adenocarcinoma	101	92.7%
Serous/Clear cell carcinoma	3	2.8%
Others *	5	4.6%
MI (*n*)	109	
no MI	18	16.5%
MI < 50%	67	61.5%
MI ≥ 50%	24	22.0%
Grade (*n*)	109	
G1	53	48.6%
G2	33	30.3%
G3	23	21.1%
CSI (*n*)	109	
absent	97	89.0%
present	12	11.0%
LVSI (*n*)	107 ^†^	
absent	88	82.2%
present	19	17.8%

* Including carcinosarcoma, and mixed endometrioid and serous carcinoma. † There are two cases with indeterminate LVSI. FIGO, International Federation of Gynecology and Obstetrics; MI, myometrial invasion; CSI, cervical stromal invasion; LVSI, lymphovascular invasion.

**Table 3:** Differences of T1, T2, PD and ADC values between the different histopathologic subgroups of endometrial carcinoma.

Histopathologic Factor	Subgroups	T1 (msec)	T2 (msec)	PD (pu)	ADC (10^−3^ mm^2^/s)
MI	no MI	1264.1 (1123.4–1426.5)	121.1 (108.3–139.9)	87.2 (84.4–88.0)	1.179 (0.941–1.274)
	<50%	1211.9 (1187.1–1246.6)	105.8 (102.1–109.5)	85.2 (84.4–85.6)	1.000 (0.950–1.095)
	≥50%	1223.5 (1118.6–1277.1)	105.4 (97.0–115.4)	84.6 (83.9–85.6)	1.046 (0.948–1.094)
	*p* (no MI vs. MI)	0.431	**0.007**	**0.006**	**0.043**
	*p* (<50% vs. ≥50%)	0.889	0.739	0.482	0.811
Grade	G1	1202.0 (1141.7–1284.4)	105.8 (98.3–111.9)	85.6 (84.9–86.3)	1.113 (1.015–1.184)
	G2–3	1235.7 (1209.3–1256.7)	109.1 (105.2–112.4)	84.6 (84.1–85.3)	0.975 (0.932–1.043)
	*p*	0.187	0.552	0.057	**0.005**
CSI	absent	1216.0 (1200.6–1251.5)	108.4 (104.5–110.5)	85.3 (84.5–85.8)	1.049 (0.973–1.116)
	present	1211.4 (1094.4–1323.5)	104.3 (97.6–116.9)	84.9 (83.5–86.4)	1.020 (0.947–1.113)
	*p*	0.653	0.542	0.663	0.605
LVSI	absent	1210.6 (1187.1–1255.4)	108.3 (103.8–110.3)	85.3 (84.4–85.8)	1.068 (0.991–1.127)
	present	1238.8 (1152.0–1277.1)	107.7 (100.0–115.4)	85.2 (83.9–85.9)	0.947 (0.882–1.067)
	*p*	0.893	0.994	0.964	**0.020**

Measured T1, T2, PD and ADC values are represented as median (95% confidence interval). Bold *p* values represent statistical significance. PD, proton density; ADC, apparent diffusion coefficient; MI, myometrial invasion; CSI, cervical stromal invasion; LVSI, lymphovascular invasion.

## Data Availability

The data presented in this study are available on request from the corresponding author. The data are not publicly available due to ethical reasons.
